# Facilitating Mitochondrial Calcium Uptake Improves Activation-Induced Cerebral Blood Flow and Behavior after mTBI

**DOI:** 10.3389/fnsys.2016.00019

**Published:** 2016-03-08

**Authors:** Madhuvika Murugan, Vijayalakshmi Santhakumar, Sridhar S. Kannurpatti

**Affiliations:** ^1^Department of Radiology, Rutgers New Jersey Medical SchoolNewark, NJ, USA; ^2^Department of Pharmacology, Physiology and Neuroscience, Rutgers New Jersey Medical SchoolNewark, NJ, USA

**Keywords:** TBI, mitochondria, CBF, dietary, calcium uniporter, kaempferol, whisker barrel, oxidative metabolism

## Abstract

Mild to moderate traumatic brain injury (mTBI) leads to secondary neuronal loss via excitotoxic mechanisms, including mitochondrial Ca^2+^ overload. However, in the surviving cellular population, mitochondrial Ca^2+^ influx, and oxidative metabolism are diminished leading to suboptimal neuronal circuit activity and poor prognosis. Hence we tested the impact of boosting neuronal electrical activity and oxidative metabolism by facilitating mitochondrial Ca^2+^ uptake in a rat model of mTBI. In developing rats (P25-P26) sustaining an mTBI, we demonstrate post-traumatic changes in cerebral blood flow (CBF) in the sensorimotor cortex in response to whisker stimulation compared to sham using functional Laser Doppler Imaging (fLDI) at adulthood (P67-P73). Compared to sham, whisker stimulation-evoked positive CBF responses decreased while negative CBF responses increased in the mTBI animals. The spatiotemporal CBF changes representing underlying neuronal activity suggested profound changes to neurovascular activity after mTBI. Behavioral assessment of the same cohort of animals prior to fLDI showed that mTBI resulted in persistent contralateral sensorimotor behavioral deficit along with ipsilateral neuronal loss compared to sham. Treating mTBI rats with Kaempferol, a dietary flavonol compound that enhanced mitochondrial Ca^2+^ uptake, eliminated the inter-hemispheric asymmetry in the whisker stimulation-induced positive CBF responses and the ipsilateral negative CBF responses otherwise observed in the untreated and vehicle-treated mTBI animals in adulthood. Kaempferol also improved somatosensory behavioral measures compared to untreated and vehicle treated mTBI animals without augmenting post-injury neuronal loss. The results indicate that reduced mitochondrial Ca^2+^ uptake in the surviving populations affect post-traumatic neural activation leading to persistent behavioral deficits. Improvement in sensorimotor behavior and spatiotemporal neurovascular activity following kaempferol treatment suggests that facilitation of mitochondrial Ca^2+^ uptake in the early window after injury may sustain optimal neural activity and metabolism and contribute to improved function of the surviving cellular populations after mTBI.

## Introduction

Preclinical systems level studies of TBI during development can be approached using neuroimaging combined with behavioral assessment from the same subjects as currently done in younger TBI patients. As the number of children and young adults seeking care for TBI continues to increase, developmental aspects of mild to moderate traumatic brain injury (mTBI) which lead to disruption of neuronal circuits and formation of maladaptive circuits with aberrant behavioral responses (Pitkanen et al., 2009; McNamara et al., 2010; Li et al., 2015) need to be characterized. As brain activity related to behavioral tasks such as cognition, sleep, sensory processing, and motor responses result from spatially segregated neuronal assemblies (Harris, 2005), spatiotemporal mapping of stimulus-induced hemodynamic representatives of neural activity are significant for systems-level evaluation of neural plasticity (Logothetis et al., 2001). Hence functional Magnetic Resonance Imaging (fMRI) methods that enable spatiotemporal mapping of brain activity through changes in cerebral blood oxygenation, water diffusion or cerebral blood flow (CBF) are increasingly used to assess TBI patients (Kim et al., 2010; Slobounov et al., 2010; Johnson et al., 2012). Functional Laser Doppler Imaging (fLDI) is a preclinical neuroimaging method enabling high spatial resolution (100 μm) mapping of stimulus induced-neural activity represented by changes in microvascular CBF (Kannurpatti and Biswal, 2004, 2006). Accurate spatiotemporal mapping of neural events is limited by the sensitivity to microvascular signals compared to the contribution from large vessels. This ability to selectively discriminate signal change from arterioles and capillary compartments (closest in range to neural activity) is defined as functional resolution (Truong and Song, 2009). As shown by our previous studies, fLDI provides excellent functional resolution (Kannurpatti and Biswal, 2011) and complements preclinical fMRI for brain spatiotemporal mapping (Sanganahalli et al., 2013a). In this study, we used fLDI in a similar configuration that provided high microvascular specificity to detect activity close to neural ensembles (Kannurpatti and Biswal, 2011). Neural activity change in response to whisker stimulation between sham and mTBI animals was evaluated by assessing the spatiotemporal change in microvascular CBF in the somatosensory barrel field (S1_BF_), a region well characterized by electrophysiological and optical mapping studies of neuroplasticity (Welker, 1976; Diamond et al., 1993; Dowling et al., 1996; Kannurpatti and Biswal, 2011).

Secondary neuronal damage due to excitotoxic mechanisms and mitochondrial Ca^2+^ overload by increased Ca^2+^ influx is inevitable after TBI (Yokobori et al., 2014). However, the developing brain is relatively resistant to excitotoxic damage including relatively lesser excitotoxic Ca^2+^ dependent mitochondrial oxidative stress when compared to the adult (Kannurpatti et al., 2004). Hence the surviving neurons and their relatively Ca^2+^ resistant mitochondria may influence TBI prognosis during the developmental years. Human mitochondrial energy metabolism and Ca^2+^ uptake capacities diminish after TBI (Verweij et al., 2000), making the potentially surviving mitochondrial populations a critical therapeutic target (Giorgi et al., 2012). Slowing of mitochondrial Ca^2+^ uptake reduces neuronal activity *in vivo* (Mathiesen et al., 2011; Fluegge et al., 2012; Sanganahalli et al., 2013a,b) along with decreased oxidative energy metabolism (Kann et al., 2003; Mathiesen et al., 2011; Sanganahalli et al., 2013a,b). Hence we tested the impact of facilitating Ca^2+^ influx, known to boost neuronal electrical activity, and oxidative metabolism (Sanganahalli et al., 2013a), on long-term outcome after mTBI in developing rats. Kaempferol, a dietary flavonol compound and a known enhancer of the mitochondrial Ca^2+^ uniporter channel (mCU) was used to facilitate mitochondrial Ca^2+^ influx after mTBI. Kaempferol treatment improved post-injury behavioral outcomes and reduced alterations in neural circuit activity without augmenting mTBI-induced neuronal loss. The results indicate that facilitation of mitochondrial Ca^2+^ influx, known to boost ongoing neuronal electrical activity, and metabolism, may be a primary mechanism sustaining normal neural circuit functions in the surviving neuronal population leading to improved outcomes after mTBI.

## Methods

### Animals

Male Sprague-Dawley rats (23–24 days old; weighing 60–80 g) were procured from Charles River Laboratories, Wilmington, MA, USA and used in this study. Rats were housed in pairs under controlled conditions. All procedures were carried out in accordance with the institutional guidelines and approved by the Institutional Animal Care and Use Committee of Rutgers-New Jersey Medical School and ARRIVE guidelines.

### Lateral fluid-percussion injury

Lateral fluid percussion injury (FPI) is a mixed injury model (both focal and diffuse) leading to axonal, somal, and microvascular swelling. This leads to tissue distortion and axonal shearing both proximal and distal to the injury location (Lindgren and Rinder, 1965; Dixon et al., 1987; McIntosh et al., 1989). In comparison to a more focal cortical contusion injury (CCI), lateral FPI induces significant cell death in distant regions even as far as the corresponding contralateral cortex (Peterson et al., 2015), and neurophysiological changes in deeper regions such as the hippocampus (Gupta et al., 2012; Li et al., 2015; Pang et al., 2015). In the current mTBI model, lateral FPI was performed in rats when they reached an age of 25–26 days according to the procedures established previously in our laboratory (Gupta et al., 2012; Li et al., 2015). The injury age (P25-P26) in the current rat model corresponded to the developmental time range of approximately a year in humans (Sengupta, 2013) and can be classified as a developmental mTBI model.

In brief, rats were anesthetized with ketamine (80 mg/kg i.p)-xylazine (10 mg/kg i.p) and positioned on a stereotaxic frame. Ketamine provided rapid surgical plane anesthesia followed by quick recovery for the survival surgery procedure. A 3 mm craniotomy was performed on the left side of the skull −5 mm posterior to the bregma and 3 mm lateral to the sagittal suture keeping the dura intact. A Luer-Lock syringe hub was glued surrounding the exposed dura using a cyanoacrylate adhesive. After 24 h, injury was induced by attaching the Luer-Lock hub of an isoflurane-anesthetized rat to the FPI device (Virginia Commonwealth University, VA, USA). A pendulum drop delivered a brief 20 ms impact on the intact dura. The impact pressure was measured by an extra-cranial transducer and controlled between 2.0 and 2.2 atm. For sham group (*n* = 5), the rats were anesthetized and attached to the FPI device without the pendulum drop. Righting reflex recovery time was recorded after injury in all animals and was in the range of 5.352 ± 2.97 min. Sham and injured animals were monitored within their cage environment on a daily basis.

### Drug treatments

A subset of mTBI rats (*n* = 5) were administered 3 doses of kaemferol (1 mg/Kg i.p) diluted in saline containing 10% dimethyl sulfoxide (DMSO). The first dose was delivered 1 h following the FPI and the other doses on consecutive days. Another subset of mTBI-rats (mTBI + vehicle, *n* = 4) was administered 3 doses of vehicle (saline with 10% DMSO). The kaempferol dose level used (1 mg/Kg i.p) was significantly lower than the dose level (20 mg/Kg i.p) shown to have significant antioxidative effects *in vivo* (Lagoa et al., 2009). Intraperitoneal administration was adopted due to several benefits such as gradual absorption and broader time-peaks of plasma concentrations (Barve et al., 2009). Oral administration was not preferred due to concerns of intestinal microflora-related metabolism of the compound (Vissiennon et al., 2012). As the average half life of intravenous delivered kaempferol was 6 h and average lifetime 24 h (Barve et al., 2009), we designed the dosing regimen in 24 h intervals. 1 mg/Kg intravenous infusion of kaempferol can produce an estimated peak plasma concentration of ~250 μM/L, which is known to boost neural activity and oxidative metabolism *in vivo* (Sanganahalli et al., 2013a,b). Hence the intraperitoneal route of 1 mg/Kg kaempferol used would lead to plasma concentrations comparable to that achieved by human dietary intake of flavonol rich foods (DuPont et al., 2004).

### Behavioral testing

Following mTBI, all animals exhibited neurological deficits as assessed by a forelimb flexion test (data not shown). In order to access whisking-related sensorimotor impairment, a specific behavioral test namely the whisker stimulation-induced motor response (WSIMR) was performed at 1, 3, 7, 14, and 21 days following mTBI. We developed the WSIMR as a modification of the whisker-stimulated forelimb placement test (Schallert et al., 2000), since the forelimb placement responses in younger rats (23–30 days range) were not fully mature. During the WSIMR test, the animal was placed in a test cage and the whiskers on one side were gently stroked multiple times (10 stroke maximum) at a frequency ~5 Hz in a caudo-rostral direction with an applicator stick. The animal did not have any initial visual cue to the approaching stroke from the experimenter. Ten trials with an inter-trial gap of 30 s were performed and repeated 5 times on each side of the animal with a repetition gap of 2 min. Whisker deflections during the behavioral experiments were performed by a single experimenter to maintain consistency of the force, frequency, and angle of stimulation. Motor responses such as avoidance of stick and the proficiency of reaction to each stroke was noted and scored on a scale of 0–3: Active avoidance by running away = 3, quick head movement away from the stroking stick = 2, slower head movement away from the stroking stick = 1 and no reaction to stroking = 0. A cumulative score was obtained after averaging all measurements from each side of whisker stroking.

### Functional laser Doppler imaging (fLDI)

We performed initial pilot experiments in a different set of animals (not included in this study) adopting the mTBI experimental approach to enable fLDI measurements. Removal of the luer-lock cap from the skull was piloted in three animals post-injury which led to increased thickness of the skull around and adjoining the capped area. During the preparation of the thinned skull, the texture of the skull area was very flakey with presence of extra intracranial blood vessels which resulted in excessive bleeding and comparably different quality of ipsilateral-contralateral window preparations. Hence cap removal immediately after injury was discontinued. In the next pilot, we retained the cap after injury using normal cyanoacrylate adhesive strength. However, cap removal in the adult animal prior to fLDI measurements became difficult. Hence a gradual reduction of the cyanoacrylate adhesive strength was tested and an appropriate concentration enabling easy cap removal prior to the thinned skull window preparation was used in all animals included within the study.

Rats after 42–48 days following mTBI (P67-P73) and age-matched shams were anesthetized with urethane 1.2 g/kg i.p and placed on a warm pad for fLDI measurements. Animal core temperature was monitored and maintained at 37 ± 0.5°C with a rectal probe and homeothermic blankets (Baxter K-MOD100, Gaymar Industries, USA). After endotracheal intubation and mechanical ventilation, end-tidal CO_2_ (ETCO_2_) and arterial oxygen saturation (SPO_2_) were continuously monitored throughout the experimental duration (8100 Poet-Plus Vital Signs Monitor, Criticare, WI, USA). ETCO_2_ levels were maintained between 32 and 35 mmHg through appropriate ventilator adjustments. Animals received a bolus of gallamine triethiodide (250 mg/kg, i.p) after the start of ventilation to achieve muscle relaxation. To enable fLDI measurements animals were positioned on a stereotaxic frame with the scalp retracted from the frontoparietal cortex by a dorsal midline incision to expose the cranium. The Luer-Lock syringe hub implanted for the induction of mTBI was gently dislodged and removed from the skull using a pair of scissors. Inflammation was evaluated visually by ensuring that the fur growth was normal with no scalp wounds around the cap. After the midline scalp incision and cap removal for fLDI measurement, the exposed skull area beneath the cap was examined to ensure that the prior craniotomy area was dry and closed by skull and tissue growth. On inspection in almost all the rats imaged, the skull beneath the cap region had completely grown and closed at 42–48 days post-injury. There was no inflammation in any of the cap-retained animals during observations prior to the fLDI measurements. Furthermore, no inflammation was observed in two animals from which the cap fell off during the duration of housing for the experiments. The temporalis muscle was disconnected from the cranium and a window of skull area measuring 5 × 5 mm centered at −2 mm and +5 mm lateral to the bregma enclosing the somatosensory barrel field (S1_BF_) on either hemisphere was thinned to translucency using an air-cooled dental drill. Functional imaging using fLDI was carried out using a Moor LDI scanner (Moor Instruments, Sussex, UK) as described previously (Kannurpatti and Biswal, 2011). Briefly, a beam of laser from a low power (2 mW ± 20%, 632.8 nm) He-Ne source scanned the thinned skull window in a raster pattern and the scattered Doppler shifted beam was collected and focused onto a photo-detector. The laser Doppler flux signal which was proportional to the tissue perfusion/CBF at each measurement point was calculated and stored in the resulting image pixels. Distance between the scanner head and the thinned skull was 23 cm leading to an in-plane resolution of about 120 × 120 μm^2^ with fLDI images of 42 × 54 pixel matrix obtained covering a field of view of 5 × 6.5 mm^2^. Functional images were obtained from the somatosensory field of view on both hemispheres at a rate of 16 sec/image and a dead time of 4 s between each image. The current configuration was optimized for maximum microvascular specificity to detect activity close to neural ensembles (Kannurpatti and Biswal, 2011). Urethane was used due to its minimal cardiovascular effects, respiratory, spinal reflexes and confounds on neurovascular coupling (Masamoto and Kanno, 2012). Urethane also provided stable anesthesia over longer periods (several hours) (Sanganahalli et al., 2013a) and was best suited for the imaging procedure using fLDI.

### Whisker stimulation during fLDI acquisition

Whiskers from B-E rows and the stradlers α, β, γ, and δ were glued together using a masking tape of rectangular area of 0.5 × 0.6 cm^2^ to include up to 5 arcs. Whisker deflections were carried out in the caudo-rostral direction using air puffs of 15 ms duration from a pressurized air source delivering a pressure of 60 psi through a solenoid (James Long Company, Caroga Lake, NY) controlled by a A310 pulse generator (WPI instruments, Sarasota, FL). Air puffs were delivered through a short plastic tubing of length 50 cm connected to a standard 1 ml pipette tip with sufficient velocity within the duration of 15 ms that deflected the whiskers by ~40° in the rostro-caudal direction as characterized previously in our laboratory (Kannurpatti and Biswal, 2011). Deflected whiskers returned back to their original position before the delivery of the next air puff for the stimulus frequency of 5 Hz used in the current experiments. Whisker stimulation was performed on both sides of the animal in a sequential manner with contralateral whisker stimulation matched by ipsilateral fLDI measurement.

### Analysis and statistical parametric mapping of fLDI image data sets

The Moore LDI scanner acquisition program processed the blood flow related signal derived from the Doppler spectrum via an analog to digital (A/D) converter. The light intensity and the blood flux signals from the A/D converter was compensated for noise effects and color-coded in arbitrary flux units (relative perfusion units; rpu) before image reconstruction. Further data processing and statistical analysis was performed on the reconstructed image data using custom made programs developed in our laboratory (MATLAB, Natik, MA). Each experimental run contained 3 periods of whisker stimulation interspersed with 4 periods of rest representing an “ON/OFF” cycle along a block of 25 images. Experimental runs were repeated 5 times on each cortical field of view and averaged. An idealized box-car function representing the “ON/OFF” cycle of whisker stimulation was used as the reference waveform. The stimulus reference function was statistically cross correlated with the fLDI flux signal on a pixel-wise basis to determine activation. Cross correlation estimates the degree to which any two time-dependent signals are correlated. The distribution of the cross correlation coefficient throughout the image when no stimulus was presented (null distribution) was approximately a normal distribution and used to determine the threshold cross correlation coefficient value. A threshold of 1.96 times the standard deviation for the null distribution corresponding to an error probability of *P* < 0.05 was used for determining the activation threshold that corresponded to a correlation coefficient value of 0.22. Activated pixels in the functional maps were further controlled for false positives through a family-wise error control using a cluster size threshold of 0.04 mm^2^. Functional images were color coded according to the correlation coefficient values and overlaid on the gray scale baseline CBF images obtained by averaging all resting period images. The functional images (color overlay) and the baseline CBF images have identical spatial resolution.

### Histology

After the terminal fLDI experiments, rats were perfused with 4% paraformaldehyde in PBS, pH 7.4. Brains were removed and post-fixed for 24 h in the same paraformaldehyde solution. Following post-fixation, brains were cryoprotected in 30% sucrose in PBS for 24–48 h. Coronal sections (40 μm-thickness) were cut at approximately three horizontal planes with respect to the bregma (−0.1, −2.0, and −4.0 mm) using a Leica cryostat and mounted on gelatin-coated glass slides for histological analyses. The sections were stained with cresyl violet to mark the neurons. Brain tissue sections from both the injured-ipsilateral hemisphere and the uninjured-contralateral hemisphere were used for histological evaluations. To assess neuronal cell survival, six random counting frames/animal (three in each hemisphere), were considered in three distinct regions depending upon their lateral distance and depth from the injury site. (1) layer-IV of the S1_BF_ region (−0.1 mm to bregma), (2) Layer-II and III of the S1_BF_ region (−2 mm to bregma), and (3) hippocampal hilus (−4 mm to bregma). Neuronal cell bodies were counted from each frame obtained at a magnification of 40x using an oil immersion objective on an inverted optical microscope.

### Statistics

Data are presented as mean ± SEM for the behavioral results and mean ± SD for all others. WSIMR test on different time points were analyzed using ANOVA with *post-hoc* pair-wise comparisons to assess differences in performances between ipsilateral and contralateral-side. For fLDI and histology measurements, average from each group was calculated and the significant differences between the sham and treatment groups were analyzed using ANOVA followed by *post-hoc* HSD test or a paired *t*-test. A *P* < 0.05 was considered to be statistically significant.

## Results

**Figure 1:**
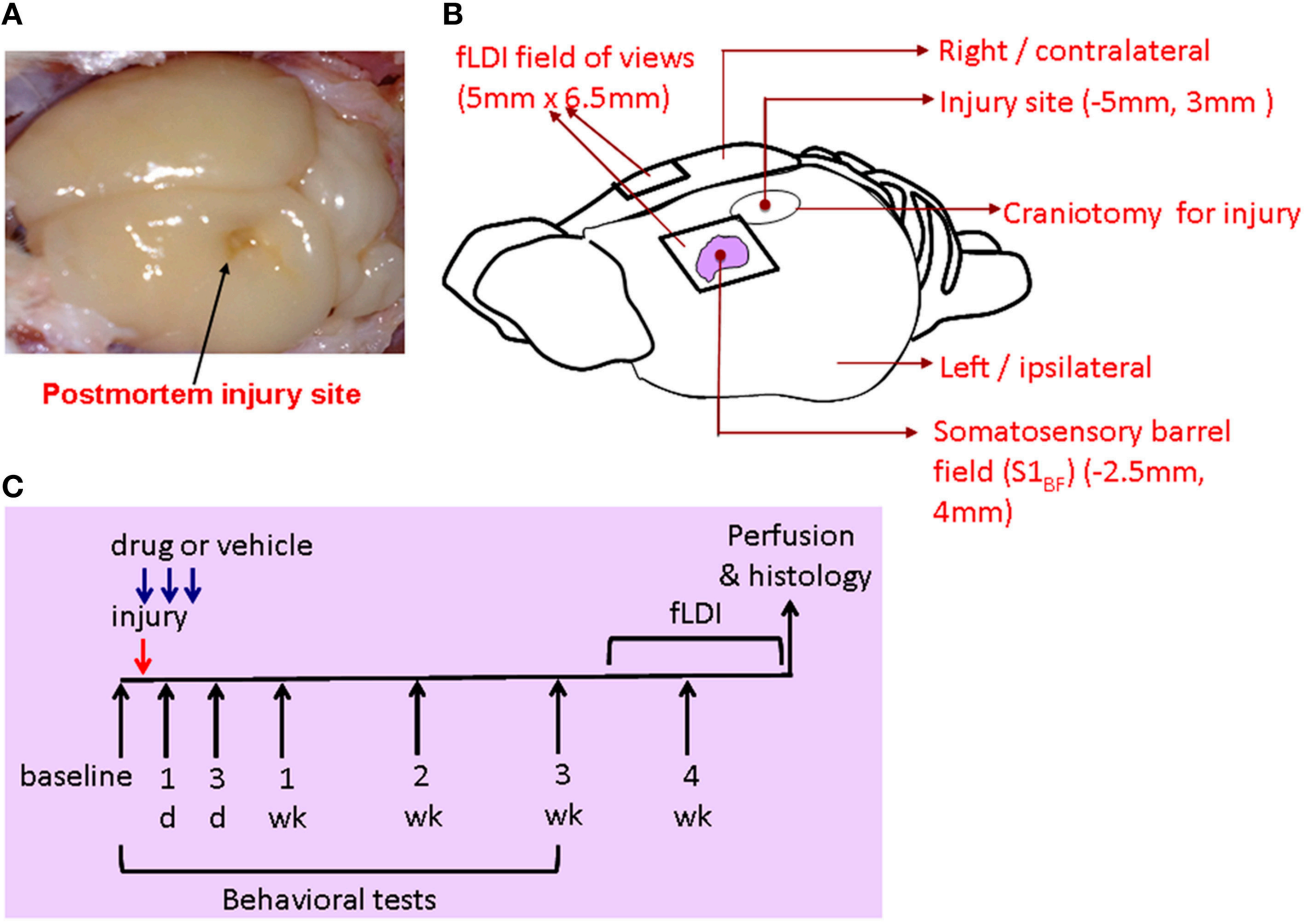
**(A)** Postmortem brain after perfusion showing the site of injury. **(B)** Schematic of the rat brain indicating the stereotaxic location of the injury and fLDI fields of view on either hemisphere covering the somatosensory whisker barrel representation. **(C)** Description of the integrated experimental design to assess mTBI prognosis after mitochondrial treatment through behavioral, fLDI, and histological markers.

Using the lateral fluid percussion rat model of TBI (Gupta et al., 2012), mTBI was performed on the hippocampal plane on the left hemisphere at −5 mm to the bregma (Figures 1A,B). This model, producing both focal and diffuse injuries, is a good representative of mild to moderate injuries in humans and well characterized in its functional impact on hippocampal (Gupta et al., 2012) and somatosensory neural circuits in rats (Lifshitz and Lisembee, 2012).

**Figure 2:**
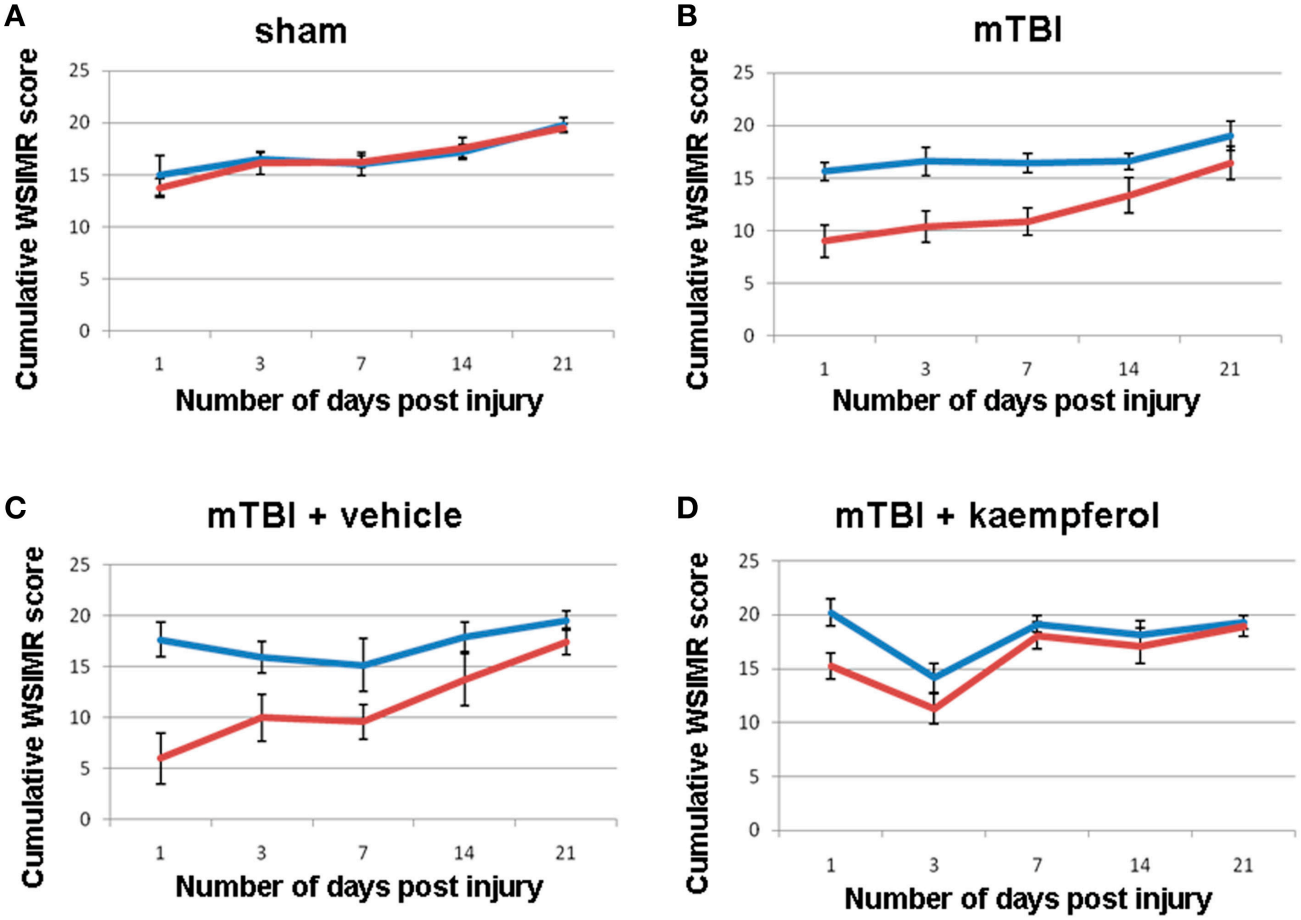
**Whisker stimulation-induced motor reactivity (WSIMR) behavioral responses in sham and mTBI animals**. **(A)** Sham group (*n* = 5), **(B)** mTBI without any treatment (*n* = 15), **(C)** mTBI animals treated with vehicle (*n* = 4), and **(D)** mTBI animals treated with kaempferol (*n* = 5). Blue curves represent WSIMR responses due to ipsilateral whisker stimulation and red curves represent WSIMR responses due to contralateral whisker stimulation. ANOVA; (*P* < 0.01; *Post-hoc* pairwise comparison) indicated significant ipsilateral-contralateral asymmetry at all behavioral time points in the mTBI and mTBI + vehicle groups. No significant difference was observed in the ipsilateral-contralateral asymmetry for all time points in the sham whereas the 1 and 3-day time points showed significant asymmetry (*P* < 0.01) in mTBI + kaempferol group. Data points represent mean ± SEM.

Sensorimotor behavior was assessed using the WSIMR test in order to correlate the S1_BF_ circuit activity mapped using fLDI. Scores were assigned depending upon the vigor of face withdrawal when the whiskers on each side were stroked. In sham animals, a progressive improvement was observed in the WSIMR scores as rats reached a young-adult age of 1.5 months (Figure 2A). In mTBI animals, there was a significant ipsilateral-contralateral asymmetry in addition to a time-dependent improvement in WSIMR scores (Figure 2B). Ipsilateral-contralateral asymmetry in behavioral performance was significant at all time points up to 21 days post-injury (Figure 2B) without any change in contralateral performance when compared to sham (Figures 2A,B; ANOVA). As WSIMR testes higher order cortical functional integration requiring the engagement of sensorimotor circuits to elicit behavioral responses, the progressive decrease in the ipsilateral-contralateral asymmetry during the 21 day period after injury indicates that recovery in higher order brain functions may continue through the late stage (>21 days post-injury). In two additional groups of mTBI animals, one was treated with the mitochondrial Ca^2+^ uniporter mCU channel enhancer, kaempferol, during the early window (0–3 days post injury) while the other received vehicle treatment. Kaempferol (1 mg/Kg, i.p) or vehicle (saline with 10% DMSO) treatments were performed at 1 h, 24 h, and 48 h after injury. Behavioral assessment revealed that kaempferol treatment significantly improved WSIMR scores after and abolished the ipsilateral-contralateral asymmetry (Figure 2D; ANOVA) compared to vehicle treated rats which showed persistent statistically significant ipsilateral-contralateral asymmetry (Figure 2C; ANOVA, *P* < 0.01; *Post-hoc* Tukey's HSD test). The brief 3 day post-injury kaempferol treatment was able to completely normalize the ipsilateral-contralateral asymmetry around 14 days post-injury and thereafter (Figure 2D).

**Figure 3:**
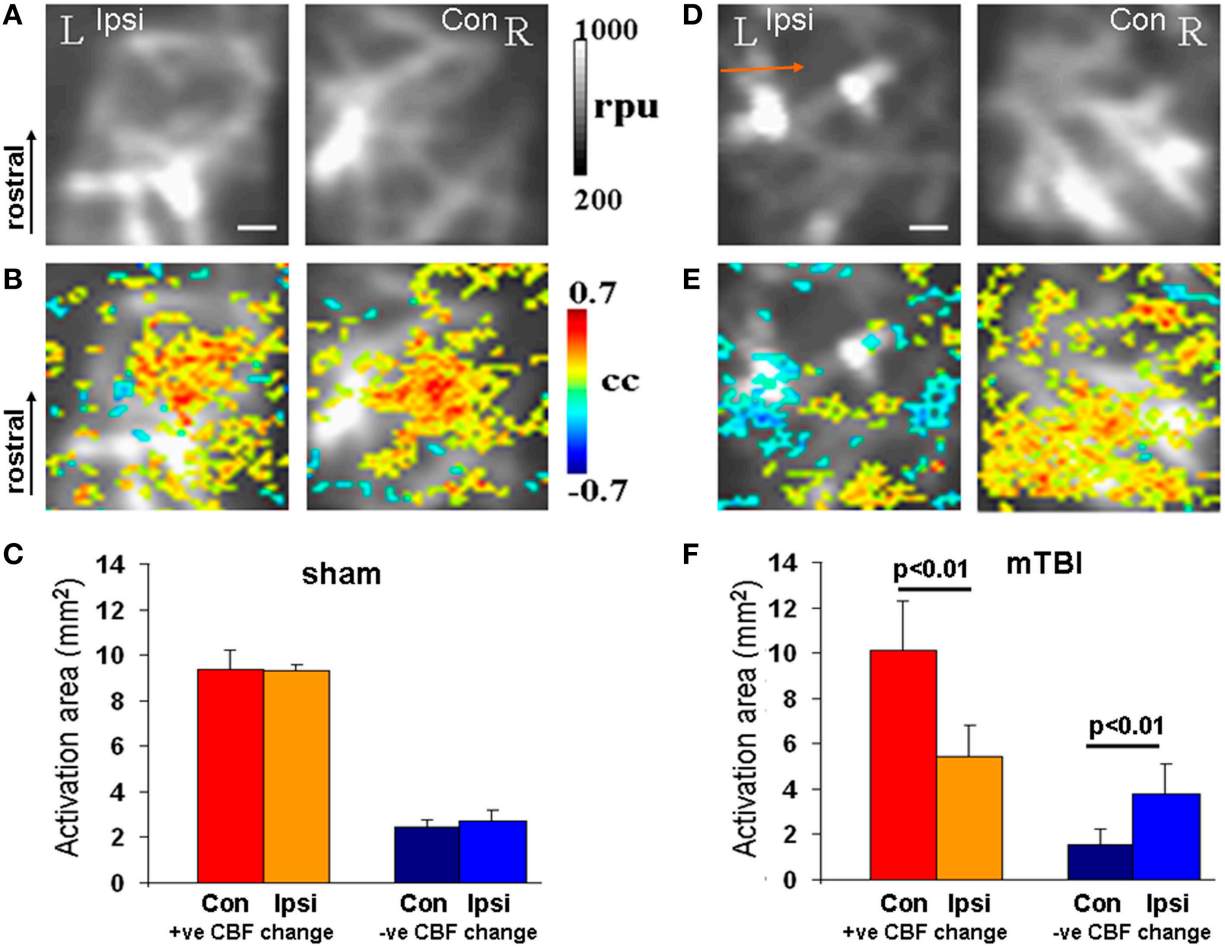
**fLDI mapping of somatosensory (S1_**BF**_) activity**. **(A,D)** Baseline CBF in sham and mTBI animals respectively showing ipsilateral decrease in the S1_BF_ region in mTBI (arrow) when compared to sham. **(B,E)** Whisker deflection-induced functional activation indicating S1_BF_ CBF changes (color map) overlaid on the baseline CBF images. A functional asymmetry in mTBI can be observed, not present in the sham. **(C,F)** Activation area of positive and negative CBF responses to whicker stimulation at the group level represented as Mean ± SD. Sham group; *n* = 3 and mTBI group; *n* = 6. Significance tested using one-way ANOVA, *Post-hoc* Tukey's HSD test. Scale bar = 1 mm, R, right; L, left; Ipsi, ipsilateral; Con, contralateral; cc, correlation coefficient; rpu, relative perfusion units.

After completion of behavioral monitoring, each animal underwent functional imaging of CBF using fLDI 42–48 days post-injury (P67-P73). fLDI spatiotemporal mapping was performed during the adult stage as the immature somatosensory cortical barrel representation continues to develop exhibiting dynamic changes in metabolism, electrical activity, neural cell types, and transmitter systems which are complete and stable after 2 months of age. As detailed in the methods section, FPI models produce neuronal damage spread across a wider distance in the brain with cell death occurring even in the contralateral cortex (Pang et al., 2015; Peterson et al., 2015). The somatosensory barrel field (S1_BF_) chosen for functional imaging of brain circuit activity to correlate with whisker deflection-induced sensorimotor behavior in the intact animal model was distal to the site of impact. fLDI imaging field of view was placed on the S1_BF_ region centered −2.5 mm caudal, 5 mm lateral to the Bregma and was ~3 mm away from the site of fluid percussion impact (Figure 1). fLDI flux value in a pixel represents the product of the number of red blood cells and their velocity. Hence larger vessels with relatively higher CBF show larger fLDI flux values (>500 rpu) relative to smaller vessels such as arterioles and venuoles (<500 rpu), which can be distinguished in the baseline CBF images (Figures 3A,D). CBF change induced by underlying neural activity and oxygen metabolism forms the basis of spatiotemporal functional mapping of brain activity (Logothetis et al., 2001). Brain activation in response to a stimulus is determined by statistical parametric mapping of the hemodynamic response on a pixel-by-pixel basis. We performed statistical parametric mapping (Methods Section Analysis and Statistical Parametric Mapping of fLDI Image Data Sets) to detect whisker stimulation-induced CBF changes within the somatosensory cortex. Increases in CBF response yielded positive correlation coefficients whereas CBF decreases yielded negative correlation coefficients. Pixels with correlation coefficients ≥0.22 (positive CBF activation) or ≤ −0.22 (negative CBF activation) corresponding to a significance threshold of *P* < 0.05 were considered activated, representing the stimulus-induced cortical activation (color overlay in Figures 3B,E). Spatiotemporal mapping of hemodynamics in response to evoked stimuli can be positive or negative in direction. However, the negative hemodynamic responses cannot always be attributed to deactivation as CBF decreases can occur during various situations such as neuronal deactivation (Shmuel et al., 2002), blood diversion to an adjacent active cortical region (Harel et al., 2002; Kannurpatti and Biswal, 2004), or neurovascular uncoupling due to pathology (Schridde et al., 2008). Hence the positive and negative spatiotemporal CBF changes can be more appropriately defined as “activation” with respect to the resting state. Positive CBF responses contralateral to the injury, was similar to that observed in sham animals, but qualitatively appeared to be less focal than sham (Figures 3A–C). Small clusters of negative CBF changes in response to evoked stimulus accompanied the positive CBF responses (Figure 3B). The sparse negative CBF responses, usually in small clusters, originated from higher baseline flux pixels (over large vessels >500 rpu), and consistent with our previous fLDI mapping studies on normal rats. These small clusters of negative CBF change can be attributed to a passive upstream decrease in CBF in certain large vessels diverting blood to adjacent cortical areas (Harel et al., 2002; Kannurpatti and Biswal, 2004). Activation area was estimated as the product of pixel area and the number of activated pixels in response to whisker stimulation. In the mTBI animals, fLDI activation was asymmetric where the injured ipsilateral hemisphere's positively activated area decreased by about 50% (Figures 3D–F). A prominent negative CBF activation with large clusters of activated pixels predominantly over microvasculature with low baseline fLDI flux values (<500 rpu) was observed in the mTBI animals (Figure 3E). These larger clusters of negative CBF responses from the microvascular pixels arise due to vasoconstriction originating from suppressed neuronal activity (Devor et al., 2007) and are distinct from the small negative CBF clusters observed mostly over large vessels in sham animals. The large cluster negative CBF responses from the microvascular pixels indicated substantially reduced excitability within the S1_BF_ region ipsilateral to the injury in the mTBI animals. Such an alteration of stimulus-induced ipsilateral CBF response in mTBI animals may have multiple origins including impaired excitatory neuronal electrical activity, enhanced neuronal inhibition (Johnson et al., 2014), impaired neurovascular coupling and/or impaired blood perfusion in the injured and adjoining regions (Hayward et al., 2010). Overall, the results highlight mTBI induced neurodegeneration with sustained behavioral deficit and altered stimulus-induced spatiotemporal CBF responses indicative of increased neuronal inhibition and/or neurovascular uncoupling.

**Figure 4:**
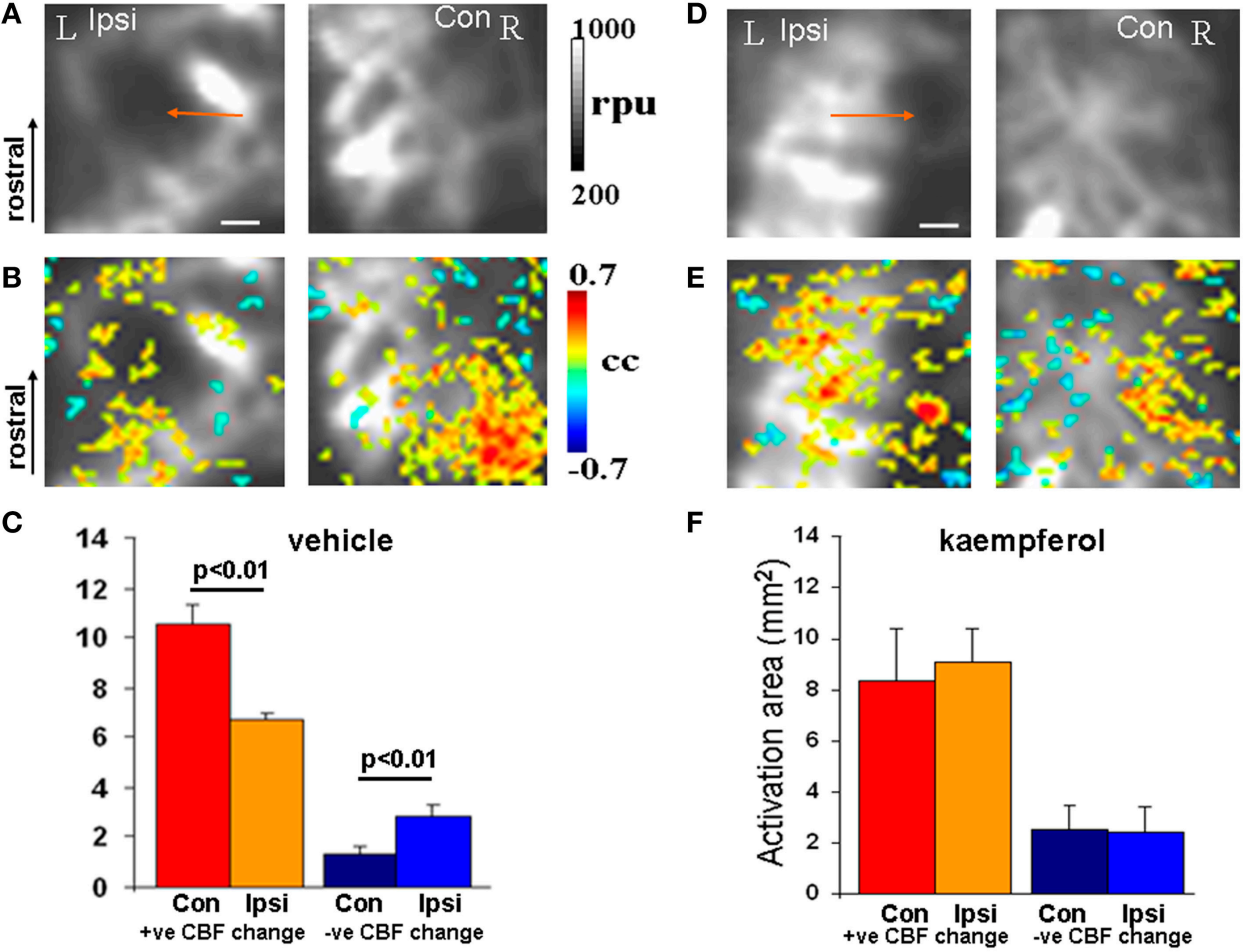
**fLDI somatosensory (S1_**BF**_) activation in mTBI animals after vehicle or kaempferol treatment**. **(A,D)** Baseline fLDI flux in the vehicle and kaempferol-treated animals respectively, indicating baseline CBF decrease in the S1_BF_ region on the injured (ipsilateral) side (arrows). **(B,E)** Whisker deflection-induced functional activation indicating CBF changes overlaid on the baseline image. A reduced functional asymmetry is evident after kaempferol treatment. **(C,F)** Activation area of positive and negative CBF responses at the group level represented as Mean ± SD. Vehicle group; *n* = 3, kaempferol group; *n* = 6. Significance tested using one-way ANOVA, *Post-hoc* Tukey's HSD test. Scale bar = 1 mm, R, right; L, left; Ipsi, ipsilateral; Con, contralateral; cc, correlation coefficient; rpu, relative perfusion units.

In separate mTBI animal groups the impact of mitochondrial Ca^2+^ uptake facilitation was assessed using fLDI after kaempferol or vehicle treatments. fLDI responses in vehicle treated mTBI animals (Figures 4A–C) was similar to that observed in the untreated mTBI animals where the stimulus-induced positive CBF response was significantly asymmetric (Figures 4A–C). Stimulus-induced positive CBF response not only improved in the kaempferol-treated animal group but the asymmetry in the ipsilateral-contralateral neurovascular activity was also significantly reduced (Figures 4D–F) compared to vehicle-treated mTBI animals (Figures 4A–C). No prominent microvascular-negative CBF responses were observed in the ipsilateral hemisphere after kaempferol treatment.

**Figure 5:**
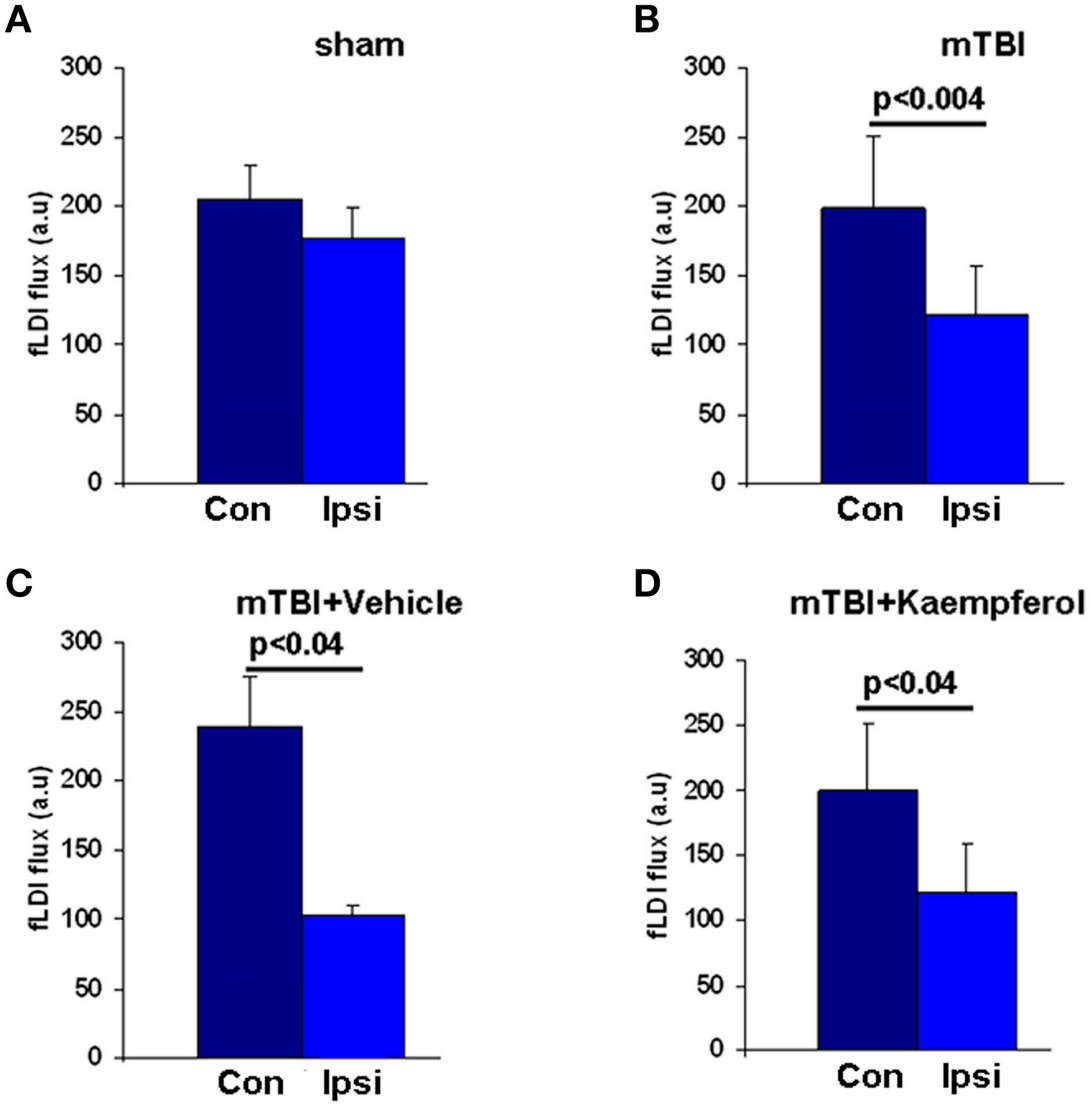
**Baseline fLDI flux representing resting CBF in all animals within the Ipsi-ipsilateral and Con-contralateral hemispheres relative to the site of injury**. **(A)** Sham, **(B)** mTBI, **(C)** mTBI animals treated with vehicle, and **(D)** mTBI animals treated with kaempferol. Data represent group level Mean ± SD, sham group; *n* = 3 and mTBI group; *n* = 6 vehicle group; *n* = 3, kaempferol group; *n* = 6. Significantly different, one-way ANOVA, *Post-hoc* Tukey's HSD test. mTBI (Ipsi) vs. (Con); *P* < 0.004 and *F* = 11.88. mTBI + vehicle (Ipsi) vs. (Con); *P* < 0.04 and *F* = 11.84. mTBI + kaempferol (Ipsi) vs. (Con); *P* < 0.04 and *F* = 6.57. No significant differences were observed in the baseline CBF levels within the same hemispheres when compared between the mTBI + vehicle and mTBI + kaempferol groups.

As brain perfusion is diminished chronically in TBI patients matching spatially with regions of neural functional deficits (Kim et al., 2010), we assessed the impact of mTBI on the baseline CBF in the developmental mTBI model used in this study. Baseline CBF was averaged over all pixels within the image field of view during the resting condition (in the absence of whisker stimulation). Baseline CBF significantly decreased across the ipsilateral cortex compared to contralateral (Figures 5A,B) accompanied by notable vascular rearrangements within the ipsilateral cortical hemisphere (Figure S1). These results are consistent with earlier MRI findings of reduced CBF accompanied by increased vascular density in the peri-lesional cortex months after the injury (Hayward et al., 2010). Kaempferol treatment, however, did not lead to any improvement in the baseline CBF decreases observed after mTBI (Figure 5D).

**Figure 6:**
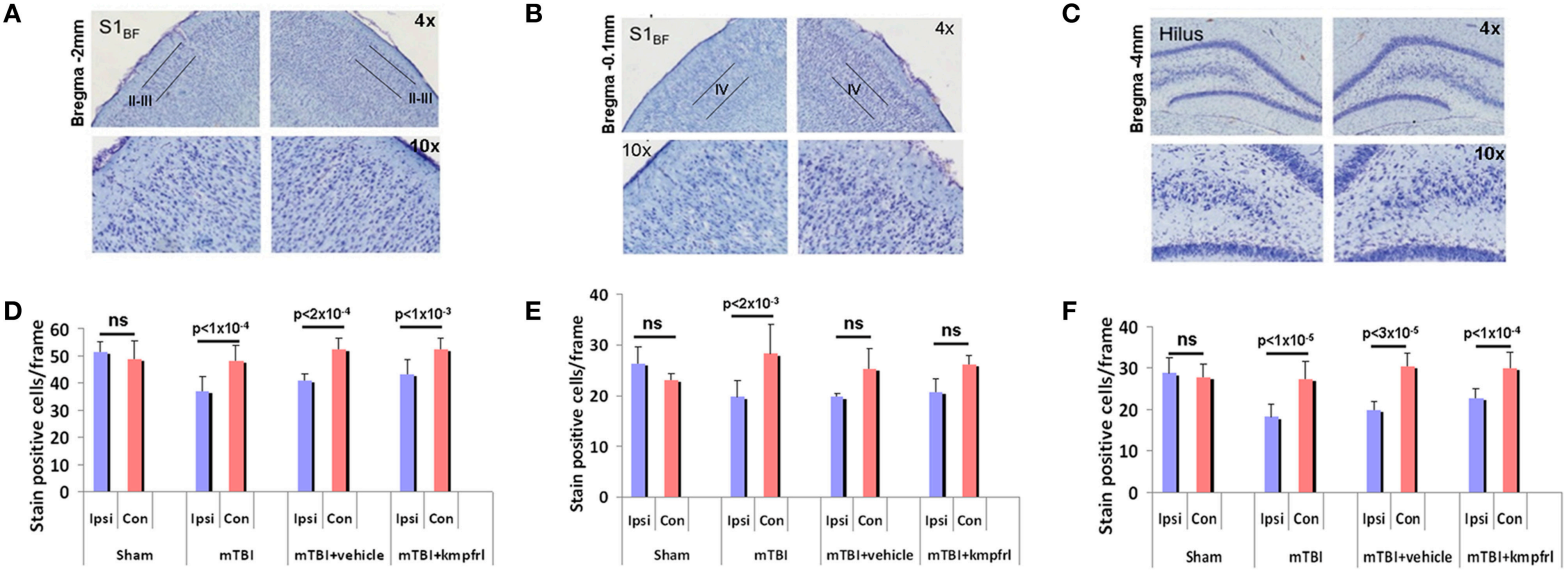
**Cresyl violet histological staining of coronal sections of post-mortem brains after terminal fLDI imaging**. **(A–C)** Representative sections over the S1_BF_ and hippocampal hilar regions in different magnifications. **(D–F)** Number of stain positive neuronal cell bodies estimated per frame from different layers of the S1_BF_ region and hippocampal hilus. Data represent group level Mean ± SD, sham group; *n* = 3 and mTBI group; *n* = 6 mTBI + vehicle group; *n* = 3, mTBI + kaempferol group; *n* = 6. Paired *t*-test comparisons between ipsilateral and contralateral neuronal counts indicated significant neuronal loss in the ipsilateral cortex and no significant protective effect of kaempferol treatment. Ipsilateral neuronal counts tested across all groups with a one-way ANOVA followed by a *post-hoc* Tukey's HSD test indicated significant differences between sham and all injured groups whereas no significant difference was observed in the contralateral neuronal counts. Kaempferol treatment after mTBI did not change neuronal survival in the adult. (S1_BF_ layer II-II; ANOVA; *F* = 9.82; Sham vs. mTBI; *P* < 0.01; Sham vs. mTBI + vehicle; *P* < 0.01; Sham vs. mTBI + kaempferol; ns; mTBI vs. mTBI + vehicle; ns; mTBI vs. mTBI + kaempferol; *P* < 0.05, mTBI + vehicle vs. mTBI + kaempferol; ns. S1_BF_ layer IV; ANOVA; *F* = 7.01; Sham vs. mTBI; *P* < 0.01; Sham vs. mTBI + vehicle; *P* < 0.01; Sham vs. mTBI + kaempferol; *P* < 0.01; mTBI vs. mTBI + vehicle; ns; mTBI vs. mTBI + kaempferol; ns, mTBI + vehicle vs. mTBI + kaempferol; ns. Hippocampal hilus; ANOVA; *F* = 20.18; Sham vs. mTBI; *P* < 0.01; Sham vs. mTBI + vehicle; *P* < 0.01; Sham vs. mTBI + kaempferol; *P* < 0.01; mTBI vs. mTBI + vehicle; ns; mTBI vs. mTBI + kaempferol; *P* < 0.05, mTBI + vehicle vs. mTBI + kaempferol; ns).

To assess neural survival after injury, coronal sections of fixed brain tissue were stained with cresyl violet to evaluate neuronal density in the distal regions such as layer IV of S1_BF_ and hippocampal hilus and S1_BF_ layers II-III stereotaxically closer to the injury impact site. Figures 6A–C show representative cresyl violet stained section of a typical mTBI brain across both hemispheres from the distal and proximal regions relative to the site of impact Average neuronal counts obtained from multiple animals indicated a significant neuronal loss within the ipsilateral hippocampal hilus (Figure 6F), typical to this injury model (Gupta et al., 2012; Li et al., 2015). Cortical layers of the S1_BF_ region distal and proximal to the injury site also indicated significant neuronal loss in the ipsilateral hemisphere compared to contralateral (Figures 6D,E). No significant difference in neuronal cell density was observed between the ipsilateral and contralateral hemispheres of sham animals. However, neuronal density in the ipsilateral cortex in all injured groups significantly reduced compared to shams (Figure 6; paired *t*-test). Kaempferol and vehicle treatments did not induce further changes in neuronal density in injured cortex either proximal or distal to the injury site (Figure 6; ANOVA).

## Discussion

Using a systems approach, the current study assessed mTBI outcomes in developing rats with clinically relevant neuroimaging markers. As mitochondrial Ca^2+^ uptake capacity is known to decrease after TBI along with impaired oxidative metabolism, neural loss, and neurovascular changes (Verweij et al., 2000; Pandya et al., 2007; Hayward et al., 2010; Lifshitz and Lisembee, 2012), we tested if mitochondrial Ca^2+^ influx facilitation, known to boost neuronal electrical activity and metabolism (Sanganahalli et al., 2013a), will impact mTBI prognosis.

Kaempferol, a dietary flavonoid compound and a specific enhancer of the mitochondrial Ca^2+^ uniporter channel (mCU) (Montero et al., 2004), capable of crossing the blood brain barrier and diffusing freely into cells and organelles (Liu et al., 2006), was administered in a dose relevant to dietary intake. Dietary therapy is recognized to be advantageous to patients recovering from mild TBI without necessitating hospitalization and intravenous access for drug administration and considered important in preclinical treatment studies of TBI (Elkind et al., 2015). Based on previous dose-dependent studies determining that 1–3 mg/Kg kaempferol delivered intravenous could enhance the baseline spontaneous/evoked neural activity and CBF responses (Sanganahalli et al., 2013a,b), a similar dose range was used with daily intraperitoneal injections of 1 mg/Kg. The current treatment can be expected to produce plasma concentrations significantly lower than 250 μM/L and comparable to ranges of kaempferol concentrations produced by human dietary intake of flavonol rich foods (DuPont et al., 2004). All animals receiving kaempferol survived with no adverse effects during the treatment which was 1000 times lower than the suggested safety limits of 1000 mg/Kg (Shih et al., 2013).

States of impaired mitochondrial Ca^2+^ uptake capacity has the potential to disrupt normal neural signaling and shown to diminish both spontaneous and activation-induced CBF and fMRI-BOLD functional responses in the working brain *in vivo* (Kannurpatti and Biswal, 2008; Sanganahalli et al., 2013a). Early post-injury brain functions are suboptimal and may coincide with the window of neuronal death/survival events (Lifshitz and Lisembee, 2012). Eelectroencephalography (EEG) and fMRI studies of humans in the early stages after mTBI indicate diminished neural circuit activity (Gosselin et al., 2011). The early-stage evolution of brain events may critically depend on mitochondrial functions within the surviving neuronal populations (Vagnozzi et al., 2007; Calderon-Cortes et al., 2008; Lifshitz and Lisembee, 2012; Sauerbeck et al., 2012; Bartnik-Olson et al., 2014). The current *in vivo* results reflect robust changes in cortical excitation where mTBI-induced brain functional activation showed decreased stimulus-evoked positive CBF responses and increased stimulus-evoked negative CBF responses (Figures 3D–F). Stimulus-evoked negative CBF responses were prominent on the injured hemisphere indicating a post-traumatic alteration in the underlying neural circuit activity or their neurovascular coupling, which may not be a desirable outcome in TBI patients. The prominent negative CBF responses in mTBI animals (large clusters of activated area) could be driven by suppressed neuronal activity within the somatosensory cortex (Shmuel et al., 2002) or neurovascular uncoupling due to pathology (Schridde et al., 2008). This is distinct from the smaller negative CBF response clusters observed in sham animals which likely reflects a passive reduction in CBF in upstream vessels of larger caliber (Kannurpatti and Biswal, 2004).

Diminished oxidative metabolism measured by oxygen utilization (indicative of mitochondrial dysfunction) has been observed in pediatric TBI patients (Ragan et al., 2013). As demonstrated by our earlier studies, diminished oxidative metabolic state achieved by inhibiting mitochondrial Ca^2+^ uptake after treatment with the mCU inhibitor Ru360 attenuated stimulus-evoked somatosensory neuronal activity and CBF responses (Kannurpatti and Biswal, 2008; Sanganahalli et al., 2013a). In contrast to Ru360, kaempferol treatment was shown to have the opposite neurophysiological effects of enhanced oxidative metabolism and neuronal activity (Sanganahalli et al., 2013a). In the current study where mTBI rats were treated with kaempferol, the brief and early mCU enhancement resulted in sustained beneficial effects eliminating inter-hemispheric asymmetry in the stimulus-evoked CBF response into adulthood. Additionally, the treatment reduced changes in neurovascular activity and activation-induced negative CBF responses across the injured ipsilateral hemisphere (Figures 4D–F). Kaempferol treatment improved somatosensory behavioral performance as early as 7 days after mTBI and the effects were sustained during the entire duration of testing up to 21 days after mTBI (Figure 2D). Hence, mitochondrial Ca^2+^ uptake impairment may be an early event within the mitochondrial populations after developmental mTBI and a potential early treatment target. Since inter-hemispheric asymmetry in somatosensory behavior was eliminated after kaempferol treatment by 7 days after mTBI and was sustained at 21 days post injury, no further behavioral testing was conducted beyond 21 days. Development of inter-hemispheric asymmetry in the spatiotemporal CBF responses measured by fLDI and restoration of inter-hemispheric symmetry by kaempferol treatment observed over 48 days after injury parallels the mTBI-induced behavioral impairment and its mitigation by kaempferol treatment 21 days after injury.

No significant neuronal survival differences were observed between kaempferol and vehicle treatments (Figure 6; ANOVA), indicating that mitochondrial Ca^2+^ uptake facilitation effects of the drug did not trigger adverse mitochondrial Ca^2+^ deregulation events including mitochondrial permeability transition. As the developing brain is relatively resistant to excitotoxic Ca^2+^ dependent mitochondrial oxidative stress and mitochondrial Ca^2+^ overload compared to the adult (Kannurpatti et al., 2004; Robertson et al., 2004), mitochondrial Ca^2+^ uptake facilitation treatments may have beneficial effects of improved neuronal activity with minimal impact on detrimental mitochondrial Ca^2+^ dependent events. Hence, mitochondrial Ca^2+^ influx facilitation in the immature brain may prove to be beneficial as it maintained optimal neuronal activity in the surviving neurons with no exacerbation of secondary neuronal death. Mitochondrial Ca^2+^ uptake enhancement also did not improve the baseline CBF decreases observed after mTBI (Figure 5D). This indicated that pharmacological facilitation of mitochondrial Ca^2+^ uptake activity might not have a significant effect on cerebrovascular cellular compartments and their functions. However, possible effects on vascular cell survival and proliferation may need further investigation as increased vascularization along with decreased CBF has been observed as a long term response in the peri-lesional cortex after mTBI (Hayward et al., 2010) and reproduced in the adulthood stage in the current studies (Figure 5 and Figure S1).

*In vivo* Ca^2+^ fluxes in the working brain in different compartments are difficult to demonstrate directly but its functional consequences can be measured *in vivo*. Impact of enhanced mitochondrial Ca^2+^ influx leads to increased mitochondrial oxidative metabolism and increased neuronal electrical activity which has been demonstrated in the intact animal model by our previous studies (Sanganahalli et al., 2013a). Hence enhanced mitochondrial Ca^2+^ uptake is the most likely event leading to altered neural functional states affected by kaempferol. Kaempferol as a dietary flavonol possesses antioxidative and anti-inflammatory effects (Middleton et al., 2000; Hamalainen et al., 2007) and may possibly impact the observed changes in brain circuit activity after treatment. Independent *in vivo* studies suggest that dose levels in the range 20 mg/Kg i.p and above are required for any significant antioxidative effects from kaempferol treatments (Lagoa et al., 2009). Although the 1 mg/Kg i.p dose of kaempferol used in this study may be suboptimal for any antioxidative effects as no neuroprotective impact was apparent in our results, non-specific antioxidative, and anti-inflammatory effects of kaempferol treatment in the developmental mTBI model cannot be excluded and needs further investigation. The current study *in vivo* tested a single compound targeting mCU activity. In order to quantify the complete physiological impact of mitochondrial Ca^2+^ uptake after developmental mTBI, similar systems level evaluation with several other mCU-targeting compounds may be required. Furthermore, stimulus-induced CBF response alterations after mTBI can have a neuronal and/or neurovascular origin. Hence future single subject electrophysiological and functional imaging correlations may be needed to determine the exact origins of the mTBI-induced functional activity changes within the brain.

In summary, suboptimal mitochondrial Ca^2+^ uptake during the early window after developmental mTBI may lead to impaired ongoing neuronal activity accompanied by neuronal loss and neurovascular coupling changes with persistent behavioral deficits. Kaempferol treatment known to facilitate oxidative metabolism and the ongoing neuronal activity improved somatosensory behavior and favorably altered the post-injury S1_BF_ neurovascular activity. The results highlight the importance of maintaining mitochondrial Ca^2+^ homeostasis in the surviving mitochondrial population in the early window (0–3 days) after mTBI. Mitochondrial Ca^2+^ influx pathway can be a viable treatment target in patients with mTBI and its physiological effects can be monitored at the systems level with clinically relevant functional imaging markers.

## Author contributions

Designed the study: SK. Performed the experiments and analyzed data: MM, SK. Contributed to laboratory resources and expertise for the animal model of Traumatic Brain Injury: VS. Wrote the manuscript: MM, SK. Edited the manuscript: MM, VS, SK.

### Conflict of interest statement

The authors declare that the research was conducted in the absence of any commercial or financial relationships that could be construed as a potential conflict of interest. The reviewer HC and handling Editor declared a current collaboration and the handling Editor states that the process nevertheless met the standards of a fair and objective review. The reviewer HC and handling Editor declared their shared affiliation, and the handling Editor states that the process nevertheless met the standards of a fair and objective review.

## Supplementary material

Figure S1 **Typical mTBI rats whose postmortem brains were sectioned and stained with cresyl violet 4–5 weeks after injury indicate significant increase in vasculature in the ipsilateral compared to the contralateral cortex proximal to the site of injury**.
