# Supplementary figures and images for: Facilitating Mitochondrial Calcium Uptake Improves Activation-Induced Cerebral Blood Flow and Behavior after mTBI
Source: Front Syst Neurosci. 2016 Mar 8;10:19. doi: 10.3389/fnsys.2016.00019 (PMC4782040; doi:10.3389/fnsys.2016.00019)

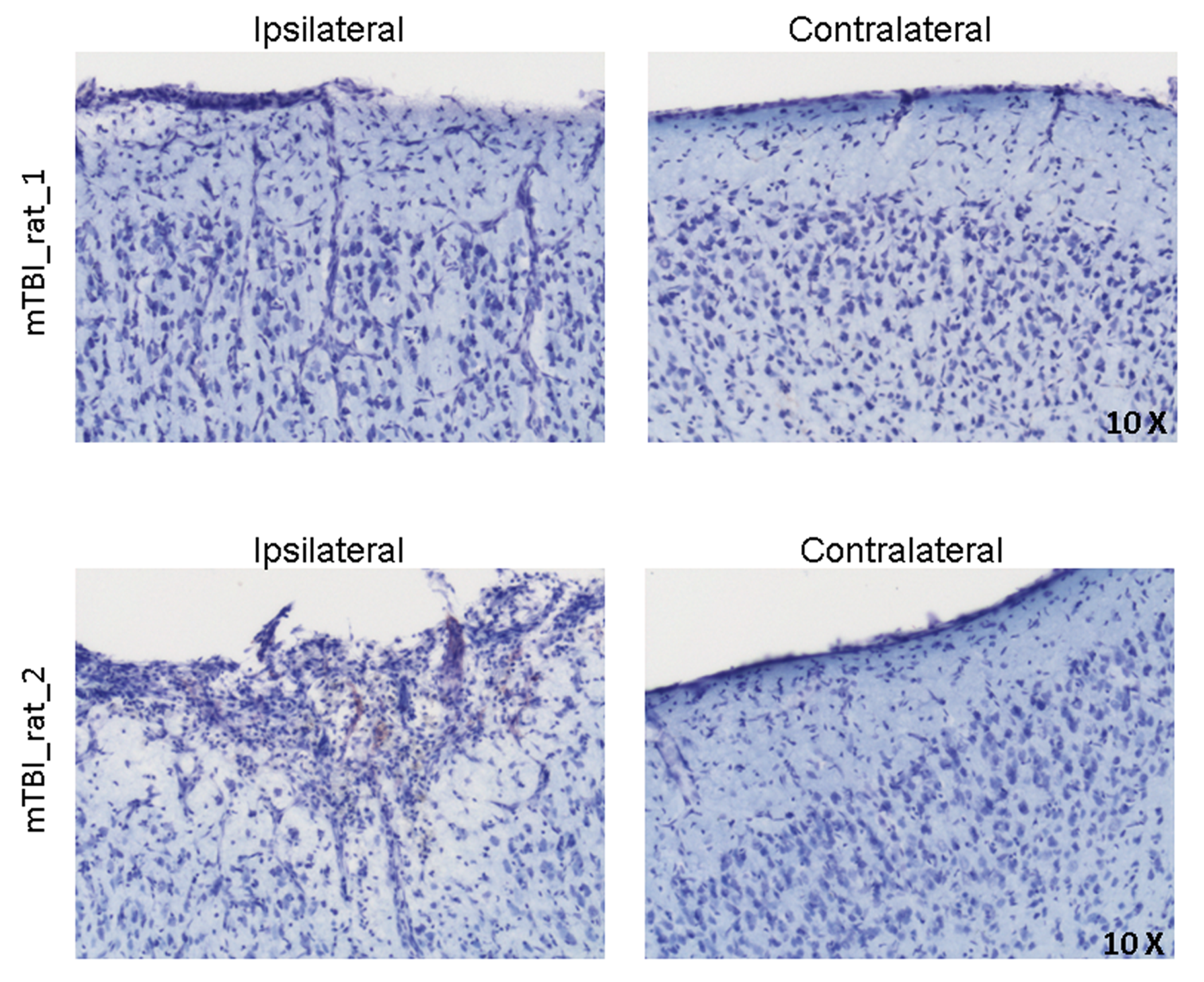

Supplement: Figure S1 — Typical mTBI rats whose postmortem brains were sectioned and stained with cresyl violet 4–5 weeks after injury indicate significant increase in vasculature in the ipsilateral compared to the contralateral cortex proximal to the site of injury. [file Image1.TIF]
